# Supplementary material for: Decoration of Anti-CD38 on Nanoparticles Carrying a STAT3 Inhibitor Can Improve the Therapeutic Efficacy Against Myeloma
Source: Cancers (Basel). 2019 Feb 20;11(2):248. doi: 10.3390/cancers11020248 (PMC6407065; doi:10.3390/cancers11020248)
Supplement: Supplementary file 1 [file cancers-11-00248-s001.pdf]

Supplementary Materials: Decoration of Anti-CD38 on Nanoparticles Carrying a STAT3 Inhibitor Can Improve the Therapeutic Efficacy Against Myeloma

Yung-Hsing Huang, Mohammad Reza Vakili, Ommoleila Molavi, Yuen Morrissey, Chengsheng Wu, Igor Paiva, Amir Hasan Soleimani, Forugh Sanaee, Afsaneh Lavasanifar and Raymond Lai

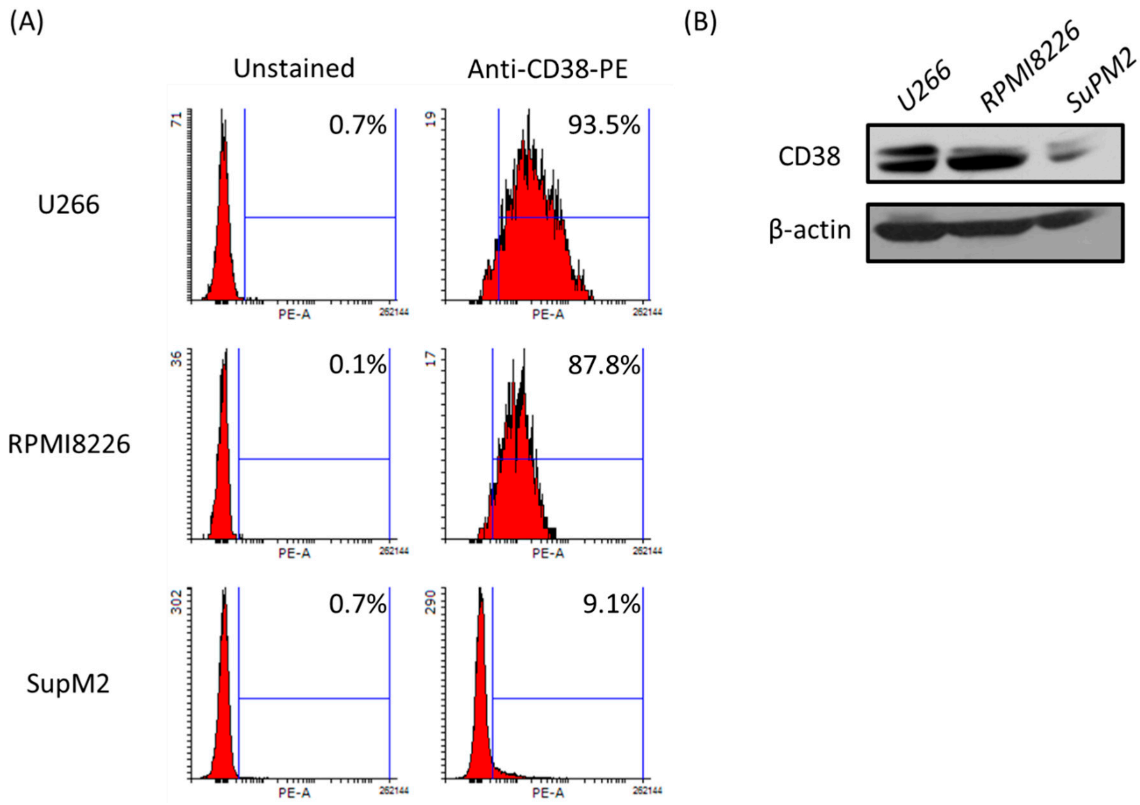

Figure S1. MM cell lines express high levels of CD38. Protein expression levels of CD38 measured by (**A**) Western blot analysis and (**B**) flow cytometry analysis in two MM cell lines (U266 and RPMI8226) and one non-MM cell line (SupM2). The gating of CD38 positivity in flow cytometry analysis was based on the unstained MM cells. β-actin was measured as a loading control for western blot analysis.

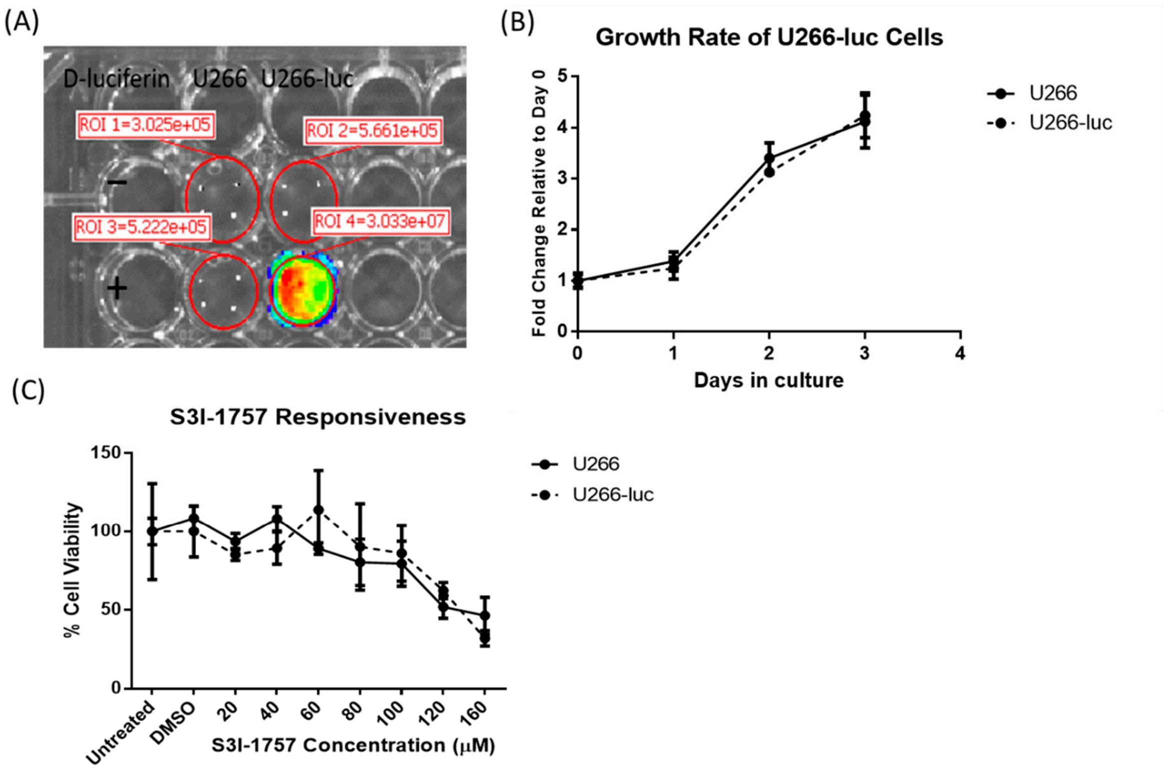

Figure S2. Characterization of U266-luc cells. (**A**) Luciferase activity in U266-luc and U266 cells was measured using bioluminescence imaging with or without the presence of D-luciferin. Total bioluminescence signal in each region of interest was quantified and shown. (**B**) The growth rate of U266 and U266-luc cells in culture over time. The number of total viable cells was measured by trypan blue exclusion assay. The cell number was normalized to day 0. (**C**) Responsiveness of U266 or U266-luc cells to S3I-1757 after 24-hour treatment. Cell viability was measured by MTS cell viability assay and normalized to the untreated cells. The experiments in (**B**) and (**C**) were carried out in triplicate and the error bar represents the stand deviation.

© 2019 by the authors. Licensee MDPI, Basel, Switzerland. This article is an open access article distributed under the terms and conditions of the Creative Commons Attribution (CC BY) license (http://creativecommons.org/licenses/by/4.0/).
